# The Traumatic Experience of Breast Cancer: Which Factors Can Relate to the Post-traumatic Outcomes?

**DOI:** 10.3389/fpsyg.2019.00891

**Published:** 2019-04-24

**Authors:** Annunziata Romeo, Marialaura Di Tella, Ada Ghiggia, Valentina Tesio, Eleonora Gasparetto, Maria Rosa Stanizzo, Riccardo Torta, Lorys Castelli

**Affiliations:** ^1^Department of Psychology, University of Turin, Turin, Italy; ^2^Clinical Psychology Unit, Città della Salute e della Scienza Hospital, Turin, Italy; ^3^Department of Neuroscience, University of Turin, Turin, Italy

**Keywords:** breast cancer survivors, post-traumatic growth, psychological distress, coping strategies, perceived social support, attachment style

## Abstract

**Background:** Post-traumatic growth (PTG) is considered a positive outcome of struggling with a traumatic event, distinct, and opposite from negative outcomes, i.e., psychological distress. The present study aimed to shed light on the relationship between potentially relating factors (i.e., coping strategies, perceived social support, and attachment style) and both positive and negative psychological outcomes.

**Methods:** A total of 123 breast cancer survivors were recruited, who completed a battery of self-report questionnaires, assessing PTG, psychological distress, coping strategies, perceived social support, and attachment style. Three regression analyses were run to evaluate whether relating factors were significant predictors of the positive and negative psychological outcomes.

**Results:** The regression analyses showed that the “Fatalism” coping strategy and perceived social support were two significant predictors of PTG. Instead, the “Helpless-Hopeless” and “Anxious Preoccupation” coping strategies, as well as an insecure attachment style, were significant predictors of depression, while the “Anxious Preoccupation” coping strategy and an insecure attachment style were significant predictors of anxiety.

**Conclusions:** The present findings showed that the factors underlying a positive or negative outcome are different and specific. While perceived social support and a fatalistic attitude seem to play a key role in the positive outcome, dysfunctional coping strategies, together with an insecure attachment style, appear to be related with negative psychological outcome. Considering these factors in clinical practice would help patients to give meaning to their traumatic experience, enhancing psychological growth.

## Introduction

Post-traumatic growth (PTG) is defined as “positive psychological change experienced as a result of the struggle with highly challenging life circumstances” (Tedeschi and Calhoun, 2004). Referring to the model of Tedeschi and Calhoun (2004), PTG is considered a positive outcome of struggling with a traumatic event, distinct from negative outcomes related to psychological distress (such as depression and anxiety).

Diagnosis of cancer is considered a type of trauma in the DSM-5 (American Psychiatric Association, 2013) and leads to many challenges. Cancer-related challenges could trigger a negative or positive reaction with different intensities depending on individual characteristics. Various factors, such as coping strategies, perceived social support, and attachment style, could contribute to and mediate the relationship between the impact of trauma and psychological outcomes. Tedeschi and Calhoun (2004) suggest that coping responses and cognitive processing to handle stressful events play a central role in the development of PTG. Coping is defined, according to the model of Lazarus and Folkman (Folkman et al., 1986), as a cognitive or behavioral effort to manage a situation evaluated as stressful. It is possible to distinguish between coping strategies associated with positive psychological outcomes, defined as “adaptive,” and coping strategies associated with negative psychological outcomes, defined as “maladaptive” (Livneh, 2000). Boyes et al. (2009), for example, found that, helpless-hopelessness, anxious-preoccupation and cognitive avoidance were maladaptive coping strategies in long-term cancer survivors. In contrast, other studies found that adaptive coping strategies, such as fighting spirit and fatalism, could enhance the PTG in patients with cancer (Sears et al., 2003; Carlsson et al., 2005; Prati and Pietrantoni, 2009; Silva et al., 2012). In particular, Silva et al. (2012) showed that in the process of coping with Breast Cancer (BC), more than one coping strategy is involved in positive changes. Women who put effort into cognitive strategies either by planning their actions, accepting life-situations, attempting to perceive BC in a more positive light, or having a humorous approach presented higher PTG.

Another factor that could be related to psychological outcomes is perceived social support. Social support, according to Schaefer and Moos (1998), may be a predictor of psychological growth by influencing coping behavior and promoting successful adaptation to life crises. Similarly, Tedeschi and Calhoun’s model of PTG (Tedeschi and Calhoun, 2004) comprises social support as a predictor of positive changes after traumatic events. However, not all the evidence is consistent. While some previous studies showed a positive association between the perception of support from significant others and the levels of PTG in cancer survivors (Cordova et al., 2001; Weiss, 2004), others found opposite results (Sears et al., 2003; Harper et al., 2007). For instance, Harper et al. (2007) found that perceived social support was not associated to positive psychological changes at three years after diagnosis.

A third factor, known to play a role in the psychological outcome after traumatic events, is attachment style. Fraley et al. (2006) found that insecure individuals reported higher levels of distress than secure ones, after the September 11 attack on the World Trade Center. Attachment styles have also been found to be associated to PTG, although limited studies have been carried on in this area and their findings are controversial (Schmidt et al., 2012; Tanyi et al., 2015; Romeo et al., 2017). Concerning breast cancer (BC) patients, the study of Romeo et al. (2017) found no significant difference in PTG between women with or without insecure attachment, while Tanyi et al. (2015) found that insecure attachment predicted fewer scores on PTG.

Thus, this study aims to elucidate the association between factors that could be associated with the way in which traumatic events are experienced (from here on referred to as “relating factors”) and psychological outcomes. The main purpose was to examine the relationship between relating factors (i.e., coping strategies, perceived social support, and attachment style) and positive psychological outcomes (i.e., PTG), and between relating factors and negative psychological outcomes (i.e., psychological distress). As far as we know, this study represents the first attempt to analyse, in a single work, both positive and negative outcomes after cancer diagnosis, through an extensive analysis of different potentially relating factors, which can be deeply associated to the patients’ ability of managing the disease. In particular, on one hand, we hypothesize that poor social support, an insecure attachment style, and maladaptive coping strategies can be factors associated with high levels of psychological distress. On the other hand, we assume that great social support, a secure attachment style, and effective coping strategies can be factors associated with high levels of PTG.

## Materials and Methods

### Participants and Procedures

Participants were recruited from the “Clinical and Cancer Psychology Unit” of the “Città della Salute e della Scienza” Hospital of Turin, Italy. Participants eligibility criteria included being female and diagnosed with BC, at least 18 years old, able to read and understand Italian adequately, having undergone and completed treatment (chemo and/or radiotherapy) at least one year ago, not reporting any current psychiatric diagnosis or cognitive deficits, and being in good general health to perform daily activities (Karnofsky ≥ 70).

A total of 123 survivors took part to the study. Data collected included cancer-related information, demographic information, and five self-report measures: PTG evaluation, psychological distress, coping strategies, perceived social support, and attachment style. Before completing the questionnaires, participants received the following instructions: regarding both PTG and psychological distress measures, participants were asked to think about how they have been feeling in the past week (i.e., present living), while regarding coping strategies and perceived social support, participants were asked to think of how they felt during the moments of facing cancer (i.e., past experience).

All the participants gave their written informed consent to participate in the study. The study was approved by the “Città della Salute e della Scienza,” Hospital of Turin ethics committee and was conducted in accordance with the Declaration of Helsinki.

### Measures

#### Post-traumatic Growth

The Post-Traumatic Growth Inventory (PTGI) (Tedeschi and Calhoun, 1996; Prati and Pietrantoni, 2014) is a self-report instrument of positive changes after a traumatic experience. It comprises 21 items rated on a 6-point Likert-type scale and organized in five subscales: Relating to Others, New Possibilities, Personal Strength, Spirituality, and Appreciation of Life. The total PTG score ranges from 0 to 105, with high scores indicating positive growth. PTGI has been used appropriately in previous studies with cancer patients (Soo and Sherman, 2015).

It shows an excellent total internal reliability (Cronbach’s α 0.93), and an acceptable to high internal reliability for each factor (Cronbach’s α range 0.74–0.86) (Prati and Pietrantoni, 2014).

#### Psychological Distress

The Hospital Anxiety and Depression Scale (HADS) (Zigmond and Snaith, 1983; Costantini et al., 1999) is a self-report instrument for evaluating depression and anxiety levels in patients with organic disease. It comprises 14 items representing two subscales: anxiety (HADS-A) and depression (HADS-D). Each subscale consists of 7 items on a 0–3 range and score ranges from 0 to 21, with a score of 8 or more suggesting a clinically significant level of depression/anxiety symptoms.

The HADS has shown good concurrent validity, test–retest reliability and internal consistency (Cronbach’s α scores = 0.82–0.90) (Bjelland et al., 2002).

#### Coping Strategies

The Mini-Mental Adjustment to Cancer Scale (Mini-MAC) (Watson et al., 1994; Grassi et al., 2005) is a self-report instrument for evaluating five cancer-specific coping strategies: Helplessness-Hopelessness, Anxious preoccupation, Fighting spirit, Cognitive avoidance, and Fatalism. It comprises 29 items, rated on a 4-point Likert-type scale. Each subscale score ranges from 0 to 4, a higher score corresponds to a greater use of that coping strategy.

Mini-MAC has demonstrated reliability with Cronbach’s α coefficient for each domain ranging from 0.62–0.88 (Watson et al., 1994).

#### Perceived Social Support

For the assessment of perceived social support, the total score of the Italian version of the Multidimensional Scale of Perceived Social Support (MSPSS) (Zimet et al., 1988; Prezza and Principato, 2002) was employed. The MSPSS comprises 12 items, each scored on a 7-point Likert-type scale. The total score is obtained by summing the value of each individual item. High scores correspond to high levels of perceived social support (Range 0–84).

The MSPSS has shown good internal consistency (Cronbach’s α range: 0.87–0.94) and test–retest reliability (Zimet et al., 1988).

#### Attachment Style

The Relationship Questionnaire (RQ) (Bartholomew and Horowitz, 1991) measures four adult attachment styles: secure, preoccupied, dismissing avoidant, and fearful avoidant. It consists of four items rated on a 7-point Likert-type scale. The highest of the four attachment prototype ratings can be used to categorize individuals in a certain attachment style. The RQ is a measure frequently used to assess adult attachment style and has demonstrated adequate reliability and validity across cultures (Schmitt et al., 2004).

### Statistical Analysis

All the statistical analyses were conducted using Statistical Package for Social Science, version 24.0 (IBM SPSS Statistics for Macintosh, Armonk, NY, United States: IBM Corp.).

Normal distribution was assessed using indices of asymmetry and kurtosis. All the variables were normally distributed, except two clinical variables: years from diagnosis and years from the end of treatment. Descriptive analyses were run first. Second, Pearson and Spearman correlations were computed to evaluate the possible associations between positive/negative outcomes (PTG and psychological distress), relating factors (coping strategies, attachment style, and perceived social support), and demographical/clinical variables (age, educational level, years from diagnosis, and years from the end of treatment). Correlation results were assessed using α levels corrected for seven comparisons (five PTGI subscales and two HADS subscales) according to Bonferroni.

Finally, three multiple regression analyses were run to assess whether relating factors were significant predictors of the different psychological outcomes. PTG and psychological distress (anxiety and depression, separately considered) were used as dependent variables. The enter method was used to include the variables of the predictor groups. Collinearity was assessed through the statistical factor of tolerance and Variance Inflation Factor (VIF).

## Results

### Demographic and Clinical Data

Demographic and clinical characteristics are shown in Table 1. The sample had a mean age of 54.3 (SD 7.9) years, with a mean time since BC diagnosis of 3.9 (SD 3.1) years. The majority of patients were married (63.4%) and a significant number were employed (56.1%). Many patients had been treated with chemotherapy (48.8%), radiotherapy (72.4%), and hormonal therapy (74.8%) and had completed treatment, excepted for hormonal therapy, within a mean of 2.5 (SD 1.5) years. The mean score of Karnofsky (96.6 ± 6.4) indicates an excellent performance in daily activities.

**Table 1:** Demographic and breast cancer-related characteristics (*N* = 123).

	Mean (SD)	Range	Frequency (%)
Age (years)	54.3 (8.0)	32–72	
Educational level (years)	12.4 (3.9)	5–24	
Years since diagnosis	3.9 (3.1)	2–19	
Years since the end of treatment	2.5 (1.5)	1–9	
**Marital status**			
Single			16 (13)
Cohabiting			13 (10.6)
Married			78 (63.4)
Divorced			12 (9.8)
Widowed			4 (3.3)
**Work status**			
Student			1 (0.8)
Employed			69 (56.1)
Unemployed			29 (23.6)
Retired			24 (19.5)
Chemotherapy			60 (48.8)
Radiotherapy			89 (72.4)
Hormonal therapy			92 (74.8)
Karnofsky	96.6 (6.4)	70–100	


### Psychological Assessment

Data concerning the psychological assessment are shown in Table 2. Regarding HADS results, 25% patients reported clinically relevant depressive symptoms, while 37% of them reported clinically relevant anxiety symptoms.

**Table 2:** Psychological variables (*N* = 123).

	Mean (SD)	Frequency (%)	Score/100	Range
*Psychological Distress*				
HADS depression	5.1 (4.0)	31 (25.2)^#^		0–16
HADS anxiety	6.6 (4.2)	46 (37.4)^#^		0–20
*Post-traumatic growth*				
PTGI – Relating to others	16.9 (9.1)		48.3	0–35
PTGI – New possibilities	10.6 (6.6)		42.4	0–25
PTGI – Personal strength	11.5 (5.4)		57.5	0–20
PTGI – Spiritual change	3.6 (3.5)		36	0–10
PTGI – Appreciation of life	9.4 (4.4)		63.4	0–15
PTGI – Total	52.1 (23.9)		49.6	0–103
*Coping strategies*				
Mini MAC – Helpless-Hopeless	1.4 (0.5)			1–3.4
Mini MAC – Anxious preoccupation	2.1 (0.6)			1–3.9
Mini MAC – Cognitive Avoidance	2.4 (0.8)			1–4
Mini MAC – Fatalism	2.9 (0.6)			1.2–4
Mini MAC – Fighting spirit	3.0 (0.8)			1.3–4
*Perceived social support*				
MSPSS	72.7 (10.2)			42–84
*Attachment style*				
RQ – Secure	4.3 (2.0)	51 (41.8)		0–7
RQ – Dismissing	3.9 (2.2)	43 (35.2)		0–7
RQ – Preoccupied	2.5 (1.7)	9 (7.4)		0–7
RQ – Fearful	2.7 (2.0)	19 (15.6)		0–7


*PTGI, Post-Traumatic Growth Inventory; HADS, Hospital Anxiety and Depression Scale; Mini-MAC, Mini Mental Adjustment to Cancer; MSPSS, Multidimensional Scale of Perceived Social Support; RQ, Relationship Questionnaire.*

*^#^Frequency of patients over cut-off.*

Participants showed a mean PTGI total score of 52.1 (SD 23.9) (see Table 2). Following the procedure adopted in a previous study (Wang et al., 2014), PTGI scores were converted into scores out of hundred [(mean score/possible highest score)^∗^100] to compare the values of each subscale score. Participants presented the most positive level of PTG in the “Appreciation of life” subscale.

Mini-MAC scores indicated that the most common coping strategy was “Fighting spirit,” followed by “Fatalism.”

Finally, concerning MSPSS, patients reported high levels of perceived social support (maximum score 84), while in RQ, the majority reported secure attachment (42%) and dismissing attachment style (35%).

### Correlations

Results of the bivariate correlations are presented in Table 3. Results of the correlation analyses between the two psychological outcomes were not included in Table 3 for space reasons. No significant correlation was found between total PTGI and HADS-A scores (*r* = -0.078; *p* = 0.393), while a significant negative association was found between total PTGI and HADS-D scores (*r* = -0.259; *p* = 0.004).

**Table 3:** Pearson or Spearman correlations between demographic/clinical variables, psychological distress, post-traumatic growth, types of coping strategies, perceived social support, and attachment style (*N* = 123).

	HADS-D	HADS-A	PTGI – Relating to others	PTGI – New possibilities	PTGI – Personal strength	PTGI – Spiritual change	PTGI – Appreciation of life	PTGI – Total
Age	*r* = -0.090	*r* = -0.132	*r* = -0.136	***r* =** -**0.324**^∗∗^	*r* = -0.107	*r* = 0.019	***r* =** -**0.279**^∗^	*r* = -0.214
Educational level	*r* = -0.195	*r* = -0.216	*r* = 0.167	*r* = 0.216	*r* = 0.120	*r* = 0.090	*r* = 0.181	*r* = 0.197
Years since diagnosis	*r_s_* = -0.013	*r_s_* = -0.004	*r_s_* = 0.061	*r_s_* = 0.131	*r_s_* = 0.237^∗^	*r_s_* = 0.039	*r_s_* = 0.173	*r_s_* = 0.161
Years since the end of treatment	*r_s_* = -0.120	*r_s_* = -0.109	*r_s_* = -0.029	*r_s_* = 0.059	*r_s_* = 0.018	*r_s_* = -0.041	*r_s_* = 0.161	*r_s_* = 0.049
Mini MAC – Helpless-Hopeless	***r* = 0.507**^∗∗^	***r* = 0.452**^∗∗^	*r* = -0.048	*r* = -0.023	*r* = -0.135	*r* = -0.026	*r* = -0.024	*r* = -0.064
Mini MAC – Anxious preoccupation	***r* = 0.480**^∗∗^	***r* = 0.590**^∗∗^	*r* = -0.103	*r* = -0.062	*r* = -0.193	*r* = -0.098	*r* = 0.021	*r* = -0.111
Mini MAC – Cognitive Avoidance	*r* = 0.169	***r* = 0.373**^∗∗^	*R* = -0.111	*r* = -0.137	*r* = -0.019	*r* = 0.021	*r* = 0.075	*r* = -0.068
Mini MAC – Fatalism	*r* = -0.213	*r* = -0.050	*r* = 0.223^∗^	*r* = 0.146	*r* = 0.174	***r* = 0.256**^∗^	***r* = 0.243**^∗^	***r* = 0.251**^∗^
Mini MAC – Fighting spirit	*r* = -0.180	*r* = -0.111	*r* = 0.075	*r* = 0.034	*r* = 0.060	*r* = 0.159	*r* = 0.029	*r* = 0.080
MSPSS	***r* =** -**0.365**^∗∗^	*r* = -0.230^∗^	***r* = 0.261**^∗^	*r* = 0.183	*r* = 0.219	*r* = 0.058	*r* = 0.193	***r* = 0.244**^∗^
RQ – Secure	***r* =** -**0.313**^∗∗^	***r* =** -**0.255**^∗^	*r* = 0.150	*r* = -0.008	*r* = 0.087	*r* = 0.068	*r* = 0.070	*r* = 0.097
RQ – Dismissing	*r* = 0.087	*r* = 0.55	*r* = -0.103	*r* = -0.081	*r* = 0.043	*r* = -0.024	*r* = -0.090	*r* = -0.072
RQ – Preoccupied	***r* = 0.308**^∗^	***r* = 0.350**^∗∗^	*r* = -0.035	*r* = -0.041	*r* = -0.087	*r* = 0.006	*r* = 0.085	*r* = -0.028
RQ – Fearful	*r* = 0.193	*r* = 0.205	*r* = -0.040	*r* = 0.099	*r* = 0.006	*r* = -0.049	*r* = 0.119	*r* = 0.028


PTGI, Post-Traumatic Growth Inventory; HADS-A and HADS-D, Anxiety and Depression subscales of the Hospital Anxiety and Depression Scale; Mini-MAC, Mini Mental Adjustment to Cancer; MSPSS, Multidimensional Scale of Perceived Social Support; RQ, Relationship Questionnaire. ^*^p < 0.007 (i.e., the critical p-value after Bonferroni correction). ^**^p < 0.001. Significant correlations have been highlighted using bold font.

#### Positive Outcome: Post-traumatic Growth

As shown in Table 3, PTG was not significantly related to demographic and clinical variables, except for the subscales “New possibility” (*p* < 0.001) and “Appreciation of life” (*p* = 0.002), which were negatively related to age. Among different coping strategies, only “Fatalism” was significantly associated with both the total PTGI score (*p* = 0.005), and the subscales “Appreciation of life” (*p* = 0.007) and “Spiritual change” (*p* = 0.004) scores.

Concerning attachment style, no significant correlation was found with any of the PTGI scores. Finally, positive correlations were found between MSPSS and both the total (*p* = 0.007) and “Relating to others” subscale (*p* = 0.004) PTGI scores.

#### Negative Outcome: Anxiety and Depression

No significant correlation was found between HADS-A and D, and the demographic and clinical variables (Table 3).

With respect to coping strategies, significant correlations were found between Mini-MAC and psychological distress. A greater use of “Helpless-Hopeless” and “Anxious preoccupation” coping strategies positively correlated with both HADS-D (*p* < 0.001) and HADS-A (*p* < 0.001). However, “Cognitive avoidance” coping strategy was positively associated only with HADS-A (*p* < 0.001).

Regarding perceived social support, a negative correlation was found between MSPSS and HADS-D (*p* < 0.001) scores.

Finally, secure attachment style was negatively related to both depression (*p* < 0.001) and anxiety (*p* = 0.005) symptoms, while preoccupied attachment was positively related to both HADS-D (*p* = 0.001) and HADS-A (*p* = 0.005).

### Multiple Regressions

#### Positive Outcome: Post-traumatic Growth

A multiple regression analysis was conducted first to examine the relative contribution of coping strategies and perceived social support to the PTG (Table 4). Since demographic, clinical, and attachment variables did not correlate with the dependent variable, they were no longer included in the regression analyses.

**Table 4:** Multiple regression predicting post-traumatic growth from types of coping strategies and perceived social support (*N* = 123).

Predictor variables	*B*	β	*t*	*R^2^*	Adj *R^2^*	*F*
*PTGI – Total*						
*Model*				0.10	0.09	6.60^∗∗^
Mini MAC – Fatalism	8.345	0.208	2.33^∗^			
MSPSS	0.477	0.199	2.23^∗^			


PTGI, Post-Traumatic Growth Inventory; Mini-MAC, Mini Mental Adjustment to Cancer; MSPSS, Multidimensional Scale of Perceived Social Support. ^*^p < 0.05; ^**^p < 0.01.

The full model of coping and perceived social support to predict the total score of PTGI was statistically significant *R^2^*= 0.10, *F*(2,118) = 6.60; *p* = 0.002; adjusted *R^2^*= 0.09. The “Fatalism” subscale of the Mini-MAC and the total score of the MSPSS explained the 9% of the variance of the PTGI total score.

Both “Fatalism” subscale of the Mini-MAC (β = 0.21; *p* = 0.022) and MSPSS (β = 0.20; *p* = 0.027) were found to be significant predictors of the model.

#### Negative Outcome: Anxiety and Depression

Two additional multiple regression analyses were run to explore the relative contribution of coping strategies, perceived social support, and attachment styles to psychological distress (Table 5). Since the demographic and clinical variables did not correlate with the dependent variables, they were no longer included in the regression analyses.

**Table 5:** Multiple regressions predicting anxiety and depression from types of coping strategies, perceived social support, and attachment style (*N* = 123).

Predictor variables	*B*	β	*t*	*R^2^*	Adj *R^2^*	*F*
*HADS Depression*					
Model				0.42	0.39	16.21^∗∗^
Mini MAC – Helpless-Hopeless	2.067	0.262	2.82**			
Mini MAC – Anxious Preoccupation	1.635	0.271	2.98**			
MSPSS	-0.039	-0.102	-1.26			
RQ – Secure	-0.389	-0.197	-2.57*			
RQ – Preoccupied	0.326	0.143	1.93			
*HADS Anxiety*					
Model				0.41	0.38	16.05^∗∗^
Mini MAC – Helpless-Hopeless	-0.152	-0.021	-0.22			
Mini MAC – Anxious Preoccupation	2.944	0.477	4.48**			
Mini MAC – Cognitive Avoidance	0.388	0.075	0.90			
RQ – Secure	-0.353	-0.162	-2.23*			
RQ – Preoccupied	0.609	0.250	3.39**			


HADS, Hospital Anxiety and Depression Scale; Mini-MAC, Mini Mental Adjustment to Cancer; MSPSS, Multidimensional Scale of Perceived Social Support; RQ, Relationship Questionnaire. ^*^p < 0.05; ^**^p < 0.01.

Concerning HADS-D, the full model of coping, perceived social support, and attachment style to predict depression was statistically significant, *R^2^*= 0.42, *F*(5,113) = 16.21; *p* < 0.001; adjusted *R^2^*= 0.39. The predictor variables were able to explain the 39% of the variance of the HAD-D. In particular, the subscales “Helpless-Hopeless” (β = 0.26; *p* = 0.006) and “Anxious preoccupation” (β = 0.27; *p* = 0.004) of the Mini-MAC and secure attachment of the RQ (β = -0.20; *p* = 0.011) were found to be significant predictors of the model.

Finally, considering HADS-A, the full model of coping, perceived social support, and attachment style to predict anxiety was statistically significant, *R^2^*= 0.41, *F*(5,116) = 16.05; *p* < 0.001; adjusted *R^2^*= 0.38. The predictor variables explained the 38% of the variance of the HADS-A. In particular, both preoccupied attachment (β = 0.25; *p* = 0.001) and secure attachment (β = -0.16; *p* = 0.028) of the RQ, as well as the subscale “Anxious preoccupation” of the Mini-MAC (β = 0.48; *p* < 0.001), were found to be significant predictors of the model.

In all three regression analyses, the statistical factor of tolerance and VIF showed that there were no interfering interactions between the variables.

## Discussion

The current study aimed to investigate the association between relating factors and positive psychological outcomes, as well as between relating factors and negative psychological outcomes, in a group of BC survivors. The results of correlations between PTGI and HADS A-D confirmed the choice to consider the two outcomes as distinct.

Regression analyses show that each relating factor plays a specific role in explaining both the positive and negative outcomes. Coping strategies and perceived social support appear to be related with the presence of PTG, while coping strategies and attachment style seem to be related with the levels of psychological distress in our group of BC survivors.

### Positive Outcome of Breast Cancer: Post-traumatic Growth

With respect to the positive outcome, the subscale “Fatalism” of the Mini-MAC and perceived social support were two significant predictors of the total PTGI score.

The use of effective coping strategies plays a key role in the process of adjustment to cancer. Previous studies suggested that only active commitment with troubles and stressors related to the illness, and a problem-focused coping strategy significantly predicted higher levels of PTG (Schroevers and Teo, 2008; Zucca et al., 2010; Scrignaro et al., 2011). However, although problem-focused coping enables patients to deal with stressful events in a more adaptive way, other coping strategies may promote positive psychological outcomes. Previous studies with participants who experienced different traumatic events found that both acceptance and positive reframing of the event, as well as religious and fatalistic coping styles, were positively associated with PTG (Linley and Joseph, 2004; Kesimci et al., 2005; Arikan and Karanci, 2012).

Regarding perceived social support, our study found that the MSPSS was significantly related to the total growth score. Our data are consistent with previous studies, in which the availability of support from partners, family members, and friends has also been found to be associated to greater PTG in cancer survivors (Cordova et al., 2001; Weiss, 2004; Schroevers et al., 2010). In particular, a longitudinal study tried to deepen the role of social support in cancer patients, finding that patients who received more emotional support from others in the period after diagnosis (3 months after diagnosis), experienced significantly more long-term PTG (8 years after diagnosis). In contrast, patients who simply perceived family and friends to be available for emotional support did not report significantly more PTG (Schroevers et al., 2010).

With respect to attachment style, no significant association was found between PTGI and RQ. However, in a previous study, secure attachment was found to be significantly correlated with higher levels of PTG (Schmidt et al., 2012). Given this uncertain evidence, future studies are needed to elucidate the association between PTG and attachment style.

Finally, it is worth noting that other factors than coping and perceived social support may be involved in explaining the levels of PTG in cancer survivors. In fact, although both variables were found to be significant predictors of the PTGI total score, they were able to explain only a small proportion of variance of the psychological growth. Such factors can include, for instance, emotional sharing and cognitive processing. In particular, Tedeschi and Calhoun (2004) suggested that the rumination processing and the questioning of core beliefs seem to be associated with psychological growth.

### Negative Outcome of Breast Cancer: Psychological Distress

Regarding the negative outcomes, we evaluated the relationships between the relating factors and depression and anxiety symptoms.

With respect to depression, the predictor variables were able to explain the 39% of the variance of the HAD-D. This result suggests that the factors we considered have an additive effect in enhancing the levels of depressive symptoms in our group of BC survivors.

In particular, both “Helpless-Hopeless” and “Anxious preoccupation” subscales of the Mini-MAC, as well as secure attachment of the RQ, were significant predictors of the HADS-D. In line with our results, it has been suggested that anxious/preoccupation and helpless/hopelessness coping strategies are associated with high levels of depression (Alcalar et al., 2012). Hopelessness represents a risk factor for depression and negatively relates to coping with BC cancer (Brothers and Andersen, 2009). Contrary to dysfunctional coping strategies, secure attachment seems to be a protective factor against the development of depressive symptoms. Our results, in accordance with those of Hamama-Raz and Solomon (2006), highlighted that secure attachment was negatively related with depression, supporting the association of a more secure attachment with greater well-being.

With regard to anxiety, the predictor variables were able to explain the 38% of the variance of the HAD-A. This result suggests that the factors we considered have an additive effect in enhancing the levels of anxiety symptoms in our group of BC survivors.

In particular, significant predictors of HADS-A were both preoccupied attachment and secure attachment styles of RQ, as well as the “Anxious preoccupation” subscale of Mini-MAC. Cicero et al. (2009), consistent with our results, found that attachment anxiety was associated with anxious preoccupation coping style in cancer patients. Similarly, Porter et al. (2012) found that patients high in attachment anxiety had significantly higher levels of anxiety. More generally, attachment anxiety was found to be a solid positive predictor of psychological distress, along with neoadjuvant treatment, in women with BC (Favez et al., 2016). Taken together, these results let us speculate that women characterized by anxiety traits (both attachment and coping style) may experience high levels of anxious symptomatology, with consequent negative outcomes. In contrast, secure attachment style appears to decrease anxiety symptoms, similar to what has been found for depression. Not surprisingly, secure attachment is a protective factor against the development of anxiety symptoms. While individuals with insecure attachment are organized by regulations that direct attention toward distress in a hypervigilant and dysfunctional way, individuals with secure attachment style can express their own emotions more, with consequent greater abilities in regulating emotional experience (Ávila et al., 2015).

Finally, perceived social support was not a significant predictor of either anxiety or depression. No correlation was found between MSPSS and HADS-A, while an initial negative correlation found between MSPSS and HADS-D was not supported by the regression analysis. Most studies on the contribution of social support in psychological adjustment to cancer have concentrated on negative outcomes mostly in the first year after diagnosis, suggesting that perception of support is related consistently to lower concurrent distress (Alferi et al., 2001; Andreu et al., 2012). Little evidence is available on the relationship between perceived social support and psychological distress in long-term cancer survivors. Future longitudinal studies are needed to investigate the association between social support and psychological distress in cancer sufferers, considering both extent and quality of support.

### Study Implications

The findings of the present study may have important implications for clinical practice. Shedding light on psychological and personological factors that can be associated with patient outcomes may allow psychologists to orient themselves in the clinical choices. The current results suggest, in fact, that the relating factors we considered are significantly and specifically associated to positive and negative outcomes after cancer diagnosis.

When developing psychological interventions for cancer patients, it is thus necessary to consider the role of these factors to decrease the levels of psychological distress and improve PTG over time. With this in mind, psychological growth can be seen as a chance of giving meaning to a traumatic event, such as cancer diagnosis, which may heavily affect one’s own life.

### Limitations

The present study has also some limitations. First, the use of self-reported instruments might have led patients to under-report or exaggerate the severity of their symptoms to minimize or exacerbate their problems. In particular, although many studies confirmed the validity of the HADS, others (Coyne and van Sonderen, 2012) pointed out that it is not a dependable instrument of differentiating anxiety and depression. Performance-based instruments or structured interviews should be employed in addition to traditional self-reported measures.

Second, cross-sectional studies do not permit certain conclusions about a causal direction to be drawn. We tried to overcome this problem by providing our BC survivors with different instructions (i.e., thinking of present vs. past experience) before completing the questionnaires. We are aware that administering some of the questions in a retrospective way may have affected the answers provided by the patients due to memory biases. However, in the recollection of a traumatic event, a central role is also played by the subjective interpretation of the event’s meaning and the emotional reaction to it (Green, 1990). Therefore, it is reasonable to assume that the patients’ answers to the questionnaires can be considered the individuals’ “real” experience of the event, as they experienced and subsequently processed it.

Third, the high number of variables included in the analyses requires caution in interpreting our results due to the cross-sectional nature of the study. Longitudinal studies assessing the main relating factors in the acute period, just after cancer diagnosis, are therefore needed to investigate their predictive value on long-term psychological outcomes.

Finally, it should be noted that our sample was recruited from a single Hospital Unit and may not be representative of the population.

## Conclusion

To the best of our knowledge, the current study has tried to examine, on a sample of 123 participants-survivors from the BC for the first time in a single work, both positive and negative outcomes after cancer diagnosis, through an extensive analysis of different potentially relating factors, which can be deeply associated to patients’ ability to manage the disease.

The present findings highlighted a different role of these relating factors in explaining positive and negative psychological outcomes, separately considered.

Fatalistic coping strategies and perceived social support seem to enhance the levels of PTG in BC survivors. In other words, patients who experienced more social support and coped with their traumatic event through a fatalist attitude managed their relationships better and reported more growth after cancer.

Maladaptive coping strategies and insecure attachment seem to be associated with the levels of psychological distress. Specifically, preoccupied attachment style and use of coping strategy oriented to anxious preoccupation seem to heighten the levels of anxiety symptomatology, while helplessness/hopelessness coping strategy appears to increase depressive symptoms in the follow-up. Secure attachment style seems, instead, to be a protective factor against the development of both anxiety and depression symptoms. In other words, patients who showed insecure attachment and tended to use maladaptive coping strategies reported fewer levels of well-being after the traumatic experience.

These results indicate that the ability to adjust to cancer experience is related with patients’ intrapsychic and relational characteristics, which must be carefully considered in order to carry out a patient-tailored psychological intervention.

## Ethics Statement

This study was carried out in accordance with the recommendations of “Città della Salute e della Scienza,” Hospital of Turin ethics committee, with written informed consent from all subjects. All subjects gave written informed consent in accordance with the Declaration of Helsinki. The protocol was approved by the “Città della Salute e della Scienza,” Hospital of Turin ethics committee.

## Author Contributions

AR, MDT, and LC conceived and designed the study and wrote the manuscript. AG, VT, EG, and MS collected the data. AR and MDT analyzed the data. LC and RT interpreted the data. Results and manuscript discussed and final version approved by all authors.

## Conflict of Interest Statement

The authors declare that the research was conducted in the absence of any commercial or financial relationships that could be construed as a potential conflict of interest.
